# The health care setting rather than medical speciality impacts on physicians adherence to guideline-conform anticoagulation in outpatients with non-valvular atrial fibrillation: a cross sectional survey

**DOI:** 10.1186/1471-2261-12-12

**Published:** 2012-02-29

**Authors:** Bernhard Gerber, Georg Stussi, Thomas Rosemann, Oliver Senn

**Affiliations:** 1 Clinic of Haematology, University Hospital Zurich, Raemistrasse 100, 8091 Zuerich, Switzerland; 2 Division of Haematology, Oncology Institute of Southern Switzerland (IOSI), Via Ospedale, 6500 Bellinzona, Switzerland; 3 Institute for General Practice and Health Services Research, University of Zurich, Pestalozzistrasse 24, 8091 Zuerich, Switzerland; 4 Institute of General Practice and Health Services Research, University of Zurich, Pestalozzistrasse 24, 8091 Zuerich, Switzerland

## Abstract

**Background:**

In patients with non-valvular atrial fibrillation (NVAF) at high risk for stroke guidelines consistently recommend long-term oral anticoagulation (OAC) with a vitamin K antagonist. However recommendations remain ambiguous in respect to the precise OAC initiation regimens. Based on the clinical observation, that the initiation of OAC for NVAF varies considerably in daily practice, we aimed to assess the current practice in Switzerland.

**Methods:**

Cross-sectional survey of randomly selected general practitioners, internists and cardiologists from different health care settings in an urban Swiss region that covers 1.4 million inhabitants. The main outcome measures were the preferred antithrombotic initiation regimen and long-term treatment in patients with newly diagnosed NVAF at high risk for stroke.

**Results:**

We received 226 out of 388 (58.2%) surveys. Compared to physicians working in a hospital setting (33.6% of respondents) physicians in ambulatory care reported more years of experience and claimed lower-use (never or seldom) of guidelines in general (47.6 vs. 12.2%). Regarding long-term thromboembolic prophylaxis 93.7% of all responders followed current recommendation by choosing an OAC. When focussing on guideline-consistent correct OAC initiation (either low-dose initial OAC or a combination of LMWH and OAC) adherence dropped to 60.6% with hospital physicians demonstrating a significantly higher use of guideline-conform OAC regimens (79.7 vs. 51.0%). Medical speciality in non-hospital physicians was not related to correct guideline-use. Hospital setting remained independently associated with a guideline-conform OAC initiation regimen (OR 2.8, p = 0.023) when controlled for medical speciality, physicians' characteristics and clinical experience. Problems when starting an anticoagulation treatment were seldom reported (never or seldom accounting for 94.1% of all responses).

**Conclusions:**

The guideline adherence with respect to OAC initiation regimens in NVAF was significantly lower when compared to long-term treatment and health care setting rather than medical speciality explained guideline-conform OAC initiation. The majority of the physicians did not consider the initiation of anticoagulation to be a major obstacle in outpatient care.

## Background

Even though actual guidelines provide clear-cut recommendations regarding the long-term stroke prophylaxis in non-valvular atrial fibrillation (NVAF), they tend to remain somewhat ambiguous with respect to the initiation regimens for oral anticoagulation (OAC) [1,2]. Several guidelines allow initiation with low-dose coumarins alone in patients where no immediate anticoagulation effect is warranted [1,3,4]. Nevertheless, in clinical practice many physicians in Switzerland still tend to a concomitant use of a low-molecular-weight heparin (LMWH) when starting an anticoagulation treatment. This approach takes into consideration that there is a transient hypercoagulable state after starting a coumarin therapy, with the level of the vitamin K-dependent endogenous anticoagulation factor protein C dropping rapidly compared to the prothrombogenic Factors (e.g. IX, X and prothrombin) which leads to a transient procoagulant/anticoagulant imbalance that might in turn trigger thrombotic occlusion of small vessels followed by necrosis (coumarine necrosis) [5]. The incidence of coumarine necrosis is not known, but is believed to range from 0.01 to 0.1% [6]. It is a serious clinical condition leading to therapeutic problems and often requiring surgical intervention. On the other hand there is an increased risk for bleeding complications in patients simultaneously treated with LMWH and OAC and some patients report practical problems with the administration of LMWH. Moreover, there have been concerns about LMWH-induced skin necrosis and LMWH-induced thrombocytopenia [7]. Based on the clinical observation, that the initiation regimens for OAC in outpatients with NVAF vary considerably, we conducted a survey aimed to assess current treatment practice in Switzerland. The purpose of our study was to assess the current practice regarding the initiation of OAC for NVAF in Switzerland, to determine the variables that influence guideline consistent treatment and to inquire whether initiation of anticoagulation is generally considered a clinical problem, as the latter might have an impact on the choice of e.g. a novel antithrombotic drug.

## Methods

### Study population

In 2009 we conducted a vignette-based survey on the initiation of oral anticoagulation with a postal questionnaire sent to selected physicians with various specialities in the canton of Zurich, Switzerland, a region that covers 1.4 million inhabitants. The study population was selected from physician databases provided by the local medical societies. The questionnaire was sent to a total of 388 physicians consisting of a random sample of general practitioners (n = 106), a random sample of the internists working in a medical practice (n = 93), as well as all the senior physicians working in a hospital department for internal medicine (n = 87) and all the cardiologists (n = 102). A postal reminder was sent 6 weeks after the initial questionnaire. The survey was based on voluntary participation. No financial incentives were provided by the investigators. Hence, no formal informed consent was obtained from the participating physicians. No ethical approval is required for this type of study.

### Study design

We performed a cross-sectional vignette-based survey targeting 4 major objectives: to determine current anticoagulation regimens in out-patients with non-valvular atrial fibrillation (NVAF), to determine the prevalence of guideline conform initiation of oral anticoagulation (OAC), to evaluate possible determinants (physician characteristics, health care setting) of guideline conform OAC regimens and to determine, whether physicians consider initiation of OAC to be a problem in patient care. The vignette described an outpatient with non-valvular atrial fibrillation at high-risk for stroke (CHADS_2_-score ≥ 2) requiring long-term OAC treatment (Appendix)

### Measurements

A guideline conform initiation of anticoagulation was defined based on current recommendations as (A) starting with a low dose regimen of a vitamin K antagonist (VKA) or (B) the use of a VKA loading dose combined with low-molecular-weight heparin (LMWH) in a therapeutic dose. Physician characteristics and medical experience were assessed as follow: Demographics (age, sex), medical speciality (general practitioner, internist, cardiologist), practice setting (non-hospital vs. hospital setting), medical experience including the case load of NVAF in the previous 6 months and experienced adverse events related to the OAC initiation (e.g. bleeding, thromboembolism, skin necrosis). The frequency of using guidelines in general was additionally assessed semi-quantitatively (e.g. rarely, often, always).

### Analysis

Categorical data are presented as frequencies. Chi-square statistics were used to assess crude associations between physicians' characteristics and a guideline conform OAC initiation regimen and long-term OAC use, respectively. To further investigate the independent relationship between a guideline conform OAC use and the health care setting (e.g. hospital setting vs. ambulatory care) multiple logistic regression analysis was applied.

## Results

Of the 388 questionnaires a total of 226 (58.2%) were returned and could be further analyzed. Responding rates between the various specialists in ambulatory care and hospital physicians ranged between 47% for general practitioners (GP) and 65% for general internists (GI). Out of the 226 respondents the distribution of medical speciality was as followed: 56 (24.8%) primary care physicians, 120 (53.1%) general internists, and 50 (22.1%) cardiologists (CA). Seventy six (33.6%) out of the 226 physicians worked in hospital care. Most of the hospital physicians were specialist in internal medicine (72.4%), which explained the overall high proportion of general internists with the remaining being cardiologists (25%) and general practitioners (2.6%).

Physicians' characteristics according to medical speciality and health care setting are listed in Table 1. Of the total study sample the majority of the physicians were men (79.2%), however the proportion of men significantly varied across specialities and working setting. Overall significantly less men were working in the hospital setting compared to the ambulatory care setting and the proportion of men was higher among GP's and cardiologists compared to general internists in ambulatory care. The vast majority of the responders (84.9%) reported at least 10 years of job experience with an overall professional experience that was higher in physicians working in ambulatory care compared to hospital physicians. However the case load of NVAF patients seen in the previous 6 months was higher in hospitalists compared to GP's and general internists in ambulatory care. In ambulatory care physicians the proportion of respondents who had a NVAF case load of more than 5 patients within the past 6 months was significantly higher in cardiologists compared to their colleagues in primary care and internal medicine. Overall the proportion of ambulatory care physicians (GP, IM, CA) who reported a frequent (often or always) use of guidelines was lower compared to hospitalists (53.0 vs. 86.7%, p < 0.05). However reported guideline use significantly differed across physicians' speciality in ambulatory care with cardiologist using guidelines more regularly (90.3% always or often), followed by internists (53.8%), and GP's (29.6%).

**Table 1:** Physicians' characteristics, clinical practice and experience according to speciality and health care setting

	All N = 226	Ambulatory care N = 150 (66.4%)			Hospital care N = 76 (33.6%)
		**GP** **(N = 54)**	**IM** **(N = 65)**	**CA** **(N = 31)**	

Male N (%)	179 (79.2)	50 (92.6)	49 (75.4)#	27 (87.1)	53 (69.7)*

Experience N (%)					
- 5-10 years	36 (15.1)	1 (1.9)	3 (4.6)	0 (0)	31 (40.8)
- 10-15 years	40 (16.8)	11 (20.4)	9 (13.9)	7 (23.3)	12 (15.8)
- > 15 years	162 (68.1)	42 (77.7)	53 (81.5)	23 (76.7)	33 (43.4)*

Case load (6 months)					
- none	13 (5.4)	4 (7.4)	7 (10.8)	0 (0)	2 (2.6)
- 1 - 5	139 (57.7)	44 (81.5)	48 (73.9)	11 (35.5)	28 (36.8)
- > 5	89 (36.9)	6 (11.1)	10 (15.3)	20 (64.5)§	46 (60.5)*

Use of Guidelines					
- Rarely	81 (36)	38 (70.4)	30 (46.2)	3 (9.7)	10 (13.3)
- often	108 (48)	14 (25.9)	29 (44.6)	15 (48.4)	50 (66.7)
- always	36 (16)	2 (3.7)	6 (9.2)¥	13 (41.9)§	15 (20.0)*

Adverse events during OAC initiation					
- reported N (% of physicians)	98 (43.4)	11 (20.4)	27 (41.5)	10 (32.3)	50 (65.8)**
- thromboembolism	52 (53.1)	6 (54.5)	17 (63.0)	10 (100.0)	19 (38.0)
- bleeding	48 (49.0)	2 (18.2)	5 (18.5)	2 (20.0)	39 (78.0)
- skin necrosis	31 (31.6)	3 (27.3)	14 (51.9)	0 (0)	14 (28.0)

*p < 0.05 vs. ambulatory care setting (GP, IM, CA); #p < 0.05 vs. GP and vs. CA; §p < 0.05 vs. GP and vs. IM; ¥p < 0.05 vs. GP; ** 98 out of the 226 participants (43.4%) reported a total of 131 adverse events, due to multiple responses the percentage sum exceeds 100%, physicians in the hospital setting reported significantly more adverse events compared to physicians in ambulatory care (p < 0.05)

Hospitalists reported significantly more adverse events due to OAC compared to the various specialists working in an ambulatory setting (65.8 vs. 32.0%) (p < 0.05) (Table 1). Thromboembolism was the most prevalent adverse event reported followed by bleeding complications and skin necrosis due to OAC (Table 1).

Responses to the NVAF case vignette with regard to the OAC initiation regimens as well as to the guideline adherence are listed in detail in Table 2. The OAC initiation regimens significantly differed between the ambulatory and the hospital care setting (p < 0.001). A guideline conform OAC initiation regimen (either low-dose initial OAC or a combination of LMWH and OAC) was found in 60.6% of the whole sample. A correct adherence was more frequently observed in hospitalists compared to physicians in ambulatory care (79.7 vs. 51.0%) (p < 0.001). This difference between the two health care settings persists in the subgroups of cardiologists and general internists. Cardiologists and general internists working in the hospital setting reported a correct OAC initiation regimen in 89% and 76%, respectively, which was significantly higher compared to their counterparts in ambulatory care with 58.1% and 53.1%, respectively (p < 0.05 for both comparisons). However, adherence to a guideline conform OAC initiation regimen did not differ across physicians' specialities in ambulatory care (p = 0.43). The reported adherence to a long-term OAC regimen in the whole sample was generally high (93.7%). Again, there was a statistically significant difference between hospitalists (98.7%) and physicians in ambulatory care (91.2%) (p = 0.038). No difference across physicians' specialities in ambulatory care has been observed with regard to long-term OAC use (p = 0.096).

**Table 2:** OAC initiation regimens and guideline adherence according to speciality and health care setting

	All		Ambulatory care		Hospital care
		**GP**	**IM**	**CA**	

OAC initiation regimen N (%)	221 (100)	52 (23.5)	64 (29.0)	31 (14)	74 (33.5)*
- OAC alone loading dose	39 (17.7)	9 (17.3)	14 (21.9)	7 (22.6)	9 (12.2)
- OAC alone	30 (13.6)	5 (9.6)	3 (4.7)	2 (6.5)	20 (27.1)
- OAC + LMWH prophylactic dose	34 (15.4)	13 (25.0)	10 (15.6)	6 (19.4)	5 (6.8)
- OAC + LMWH therapeutic dose	104 (47.1)	18 (34.6)	31 (48.4)	16 (51.6)	39 (52.7)
- OAC + Aspirin	4 (1.8)	2 (3.9)	2 (3.1)	0 (0)	0 (0)
- Aspirin alone	5 (2.3)	4 (7.7)	1 (1.6)	0 (0)	0 (0)
- Various	5 (2.3)	1 (1.9)	3 (4.7)	0 (0)	1 (1.4)

Guideline conform OAC initiation (%)	134 (60.6)	23 (44.2)	34 (53.1)	18 (58.19)	59 (79.7)*

Long-term OAC regimen (%)	207 (93.7)	45 (86.5)	58 (90.6)	31 (100)	73 (98.7)*

Total N = 221, 5 respondents were excluded due to multiple responses thus a sound classification of the OAC initiation regimen was not possible; * p < 0.05 vs. ambulatory care setting (GP, IM, CA)

Results of the multiple logistic regression analysis to further assess the independent association between OAC adherence and the health care setting are presented in Table 3 and 4. The hospital setting remained significantly associated with a higher guideline conform OAC initiation regimen when controlled for potential confounders (Table 3). This relationship has not been observed anymore for the long-term OAC regimen in the multivariable analysis (Table 4).

**Table 3:** Adjusted associations between a guideline conform OAC initiation in relation to the health care setting

	OR	95%-CI	p-value
**Model 1 (N = 221):**			

Ambulatory care	1.00		

Hospital care	3.5	1.7-7.0	< 0.001

**Model 2 (N = 219):**			

Ambulatory care	1.00		

Hospital care	2.8	1.2-6.9	0.023

Model 1: Adjusted association between guideline a conform OAC initiation and the health care setting controlled for physicians' speciality (GP, internist, cardiologist)

Model 2: Model 1 in addition adjusted for physicians' characteristics (gender, general use of guidelines) and clinical experience (adverse events, years of experience, NVAF cases last 6 months)

**Table 4:** Adjusted associations between a long-term OAC regimen in relation to the health care setting

	OR	95%-CI	p-value
**Model 1 (N = 221):**			

Ambulatory care	1.00		

Hospital care	5.8	0.7-49.2	0.10

**Model 2 (N = 219):**			

Ambulatory care	1.00		

Hospital care	4.8	0.4-59.5	0.22

Model 1: Adjusted association between a long-term OAC regimen and the health care setting controlled for physicians' speciality (GP, internist, cardiologist)

Model 2: Model 1 in addition adjusted for physicians' characteristics (gender, general use of guidelines) and clinical experience (adverse events, years of experience, NVAF cases last 6 months)

In the whole study sample problems when starting an anticoagulation treatment were seldom reported (never or seldom accounting for 94.1% of all responses). The cumulative prevalence of physicians reporting to often or always encounter problems related to the OAC initiation was significantly higher in the hospital setting (9.7%) compared to physicians in the ambulatory care setting (4.0%) (p for difference < 0.001).

## Discussion

The data of our survey confirm the expected variability in guideline adherence in respect to initiation of anticoagulation in non-valvular atrial fibrillation (NVAF), with health care setting rather than medical speciality being associated with a recommendation consistent treatment. The majority of the physicians does not consider the initiation of OAC to be a major problem in patient care.

The factors commonly influencing guideline adherence of physicians are well known and consist in (i) the quality of the data leading to the actual treatment recommendations, (ii) familiarity with the recommendations (iii) the physicians agreement with the recommendations, (iv) the expected impact of a therapeutic measure on the disease outcome (v) the patient population and (vi) organisational factors such as workload and convenience of the recommendations) [8,9]. We discuss the results of our survey based on these key factors.

Quality of data: In NVAF there is an imbalance in the quality of the data leading to the actual treatment recommendations. While there are well-established scoring systems assessing the risk for stroke in NVAF (e.g. CHA2DS2-VASc and CHADS2) and an effective prophylaxis with the vitamin K antagonists (VKA) leading to a class IA recommendation for long-term VKA prophylaxis in high-risk patients with NVAF, data regarding the optimal initiation of OAC is sparse [1,10-16]. E.g. there is no prospective trial comparing an OAC initiation regimen with and without concurrent heparin or low-molecular-weight heparin (LMWH) use. In addition, there is a lack of data on the incidence of heparin- and warfarin-induced skin necrosis and the occurrence of stroke when commencing an OAC prophylaxis, which leads to considerable uncertainty about the need for concurrent OAC and Heparin or LMWH. Therefore, recommendations are less stringent for OAC initiation but nevertheless, ACCP 2008 guidelines as well as BCSH 2005 guidelines do allow a low-dose VKA commencement without concurrent heparin or LMWH if no immediate anticoagulation effect is warranted [3,4]. This difference in the quality of scientific data might be reflected by our study results which confirm a high theoretical adherence to guidelines in respect to long-term OAC (93.7%) whereas a correct initiation regimen defined as choosing either a low-dose VKA scheme or adding LMWH to a VKA loading dose scheme was chosen by only 60.6% of all participants. We did not find a difference in the adherence of long-term OAC across medical specialities. This is in line with McCrory and coworkers who addressed physicians attitudes on long-term OAC for stroke prophylaxis in different clinical scenarios by means of a case-vignette which revealed no difference between primary care physicians, cardiologists and neurologists [17]. However we are not aware of studies that focused on the initiation of OAC in NVAF or the impact of the health care setting on this issue.

Familiarity and agreement with the recommendations: Another factor influencing guideline adherence is the knowledge of the recommendations themselves. An european study found, that cardiologists show a higher familiarity with heart failure guidelines when compared to internists, geriatricians, and primary care physicians [18]. Our data is more conflicting as 90.3% of all cardiologists working in outpatient care reported to use guidelines for decision making but only 58.2% actually chose a guideline conform OAC initiation regimen. Moreover, our data does not show a significant difference between the various medical specialities with regard to guideline-consistent anticoagulation for stroke prophylaxis in NVAF. It is known from the literature that practicing physicians have an attitudinalbehavioral discordance concerning their positive perception of clinical practice guidelines and the implementation of these guidelines into their own clinical practice [19]. Nevertheless, we can only hypothesize on the reasons for this imbalance in our cardiologist group: First, even though the cardiologists may usually be familiar with many guidelines, this might not be the case regarding this particular topic. Second, the physician might disagree with the guidelines and third, there might be a lack of outcome expectancy when adhering to the guidelines.

Outcome expectancy and patient selection: The hypothesis that the outcome expectancy and the patient selection might play an important role in clinical practice is supported by our data reporting a significant difference in guideline consistent behaviour, mainly depending on the health-care setting with hospital physicians showing a higher degree of guideline adherence than doctors in outpatient care. Patients with NVAF who have to be referred to a hospital are either relatively fit but will undergo cardioversion within 48 hours of onset or, which is more likely, represent a different patient subset with comorbidities or an acute condition leading to hospital admission [20]. The latter has an impact on OAC, as e.g. anticoagulation dependent skin necrosis is known to only rarely occur in otherwise healthy outpatients treated for NVAF but is mainly observed in more severely ill hospitalized patients with comorbidities [6,21-23]. Hence, doctors working in a hospital setting are exposed to a different patient population and more likely to be confronted with adverse events, an experience which might constrain them to a more guideline stringent behaviour [8]. As a matter of fact, hospital physicians in our study reported a significantly higher number of adverse events during the initial phase of a OAC treatment. In contrast, physicians working in a private practice claim to observe adverse events only in a minority of cases which might lessen the need for guidelines as they are not believed to significantly influence disease outcome.

The generally low use of guidelines in the general practitioner fraction is also noteworthy. We hypothesize that beside the difficulty in keeping up with a high level of knowledge in a large and heterogenous medical field, this might also be due to the high prevalence of multimorbide patients seen in family practice [24]. Physicians caring for multimorbide patients might consider it less reasonable to implement a therapeutical strategy solely based on the summation of single-disease recommendations as this will be far to comprehensive and might even proof to be harmful in this context [25,26].

### Limitations

First, the design of our study is a limitating factor as all observational studies always lack the power to proof causality. Second, even though our survey shows a high return rate of 58% and is therefore clearly representative for the clinical practice in an urban area in Switzerland, its results might not apply for other countries as e.g. in Switzerland the VKA of choice for patients with NVAF is phrenprocoumon which has a much longer elimination half life when compared to warfarin (140 vs. 40 hours). Most international trials are based on a VKA treatment with warfarin and even though the results might not differ substantially from a treatment with other VKAs, the conclusions obtained from a study with warfarin can, strictly spoken, not be translated to other treatment modalities. Third, a survey always assesses a theoretical adherence to therapies or treatment recommendations and despite the fact, that the majority of doctors might be familiar with current guidelines, they might not consequently translate them into clinical practice. In an observational study conducted in Basel, Switzerland, 31% of all patients with atrial fibrillation who qualified for an oral anticoagulation did not receive a VKA [27]. This finding is supported by data from many different regions worldwide [28].

## Conclusion

Our present study shows how physicians intend to start stroke prophylaxis in outpatients with NVAF in an urban region in Switzerland. Whilst the theoretical knowledge of the correct long-term treatment for these patients is excellent, compliance to guidelines with respect to the initiation of anticoagulation is not. There is a difference in guideline adherence based on the clinical setting, with hospital physicians showing a better guideline adherence when compared to their colleagues in outpatient care. Further studies are needed to address this issue and detect possible causes. We hypothesize that patient selection, outcome expectancy, and familiarity with guidelines might account for a good part of this difference. However, as with the introduction of any practice guideline, physicians need to be convinced that there exists compelling evidence from well-controlled clinical trials to justify implementation of these recommendations to the individual patient.

## Competing interests

All authors have completed the Unified Competing Interest form at http://www.icmje.org/coi_disclosure.pdf (available on request from the corresponding author) and declare: no support from any organisation for the submitted work; no financial relationships with any organisations that might have an interest in the submitted work in the previous three years; no other relationships or activities that could appear to have influenced the submitted work.

## Authors' contributions

BG and OS made primary contributions to data collection and analysis, interpretation of results, and writing of the manuscript. GS and TR helped to write the first draft. BG and OS contributed to the study conception and design. All authors revised the manuscript critically for important intellectual content, and all approved the final manuscript.

## Pre-publication history

The pre-publication history for this paper can be accessed here:

http://www.biomedcentral.com/1471-2261/12/12/prepub
